# Social disparities in exposures to neighbourhood obesogenic built environments in Czechia

**DOI:** 10.1093/pubmed/fdaf065

**Published:** 2025-06-09

**Authors:** Anna Bartoskova Polcrova, Thao Minh Lam, Hynek Pikhart, Jeroen Lakerveld

**Affiliations:** RECETOX, Faculty of Science, Masaryk University, Kotlarska 2, 611 37 Brno, Czech Republic; Department of Epidemiology and Data Science, Amsterdam University Medical Centers Vrije Universiteit Amsterdam, Meibergdreef 9, Amsterdam 1105 AZ, The Netherlands; Amsterdam Public Health, Health Behaviours and Chronic Diseases, Meibergdreef 9, Amsterdam 1105 AZ, The Netherlands; Upstream Team, Amsterdam University Medical Centers, Vrije Universiteit Amsterdam, Meibergdreef 9, Amsterdam 1105 AZ, The Netherlands; RECETOX, Faculty of Science, Masaryk University, Kotlarska 2, 611 37 Brno, Czech Republic; Department of Epidemiology and Public Health, University College London, 1-19 Torrington Place, London WC1E 7HB, UK; Department of Epidemiology and Data Science, Amsterdam University Medical Centers Vrije Universiteit Amsterdam, Meibergdreef 9, Amsterdam 1105 AZ, The Netherlands; Amsterdam Public Health, Health Behaviours and Chronic Diseases, Meibergdreef 9, Amsterdam 1105 AZ, The Netherlands; Upstream Team, Amsterdam University Medical Centers, Vrije Universiteit Amsterdam, Meibergdreef 9, Amsterdam 1105 AZ, The Netherlands

**Keywords:** obesity, environment, socioeconomics factors

## Abstract

**Background:**

Exposure to the environments with limited walkability and high density of unhealthy food outlets promotes obesity development and might cluster in disadvantaged neighbourhoods. This study examines the combined obesogenicity of urban neighbourhoods in Brno, and related socio-economic disparities.

**Methods:**

This study was conducted in Brno, the second-largest city in Czechia. The obesogenic index was calculated from 12 components of built food and physical activity environments for each of the 296 basic settlement units (BSUs) of Brno. The index ranged from 0 (low obesogenicity) to 100 (high obesogenicity). The social disparities were assessed using linear regression. Spatial clustering was assessed using the global Moran’s Index.

**Results:**

The median obesogenic index score for Brno’s 296 BSUs was 72.09 (IQR = 24.03), with higher scores in peripheral and industrial areas. Areas with higher proportion of people with university education had lower obesogenic scores of physical activity and overall obesogenic environment. Simultaneously, localities with higher unemployment exhibited lower obesogenic score in food and overall obesogenic environment.

**Conclusion:**

Areas with lower levels of obesogenicity were primarily concentrated in central locations. No clear socio-economic gradient was observed, although proportion of university-educated inhabitants and unemployment rates were both associated with lower obesogenic environment scores.

## Introduction

Obesity is one of the biggest challenges for current public health systems. The World Health Organization (WHO) estimates that 59% of adults in the European Region are living with overweight or obesity, which is one of the highest prevalence of all WHO regions.^1^ Numerous individual-level lifestyle interventions have been developed, implemented, and evaluated.^2^^,^^3^ However, despite all the public health efforts so far, we are still witnessing a steady increase in obesity burden.^1^ In recent years, it is increasingly recognized that the built environment shapes individual health behaviours, such as physical activity and sedentary behaviour through transport options or dietary patterns through the availability and diversity of food choices.^4^ Living in more walkable, less sprawled areas with accessible infrastructure for physical activity is associated with lower obesity risk.^5–10^ Conversely, obesogenic environments include sprawled, car-dependent neighbourhoods with limited healthy food options.^11–13^

Environmental characteristics do not occur randomly, and areas with higher or lower obesogenicity may cluster with socio-economic disparities.^14^^,^^15^ While some previously published studies described increased obesogenic exposures in disadvantaged neighbourhoods,^16^ others described none or the opposite relationship.^11^ The mapping of obesogenic neighbourhoods is essential for targeting interventions and developing strategies that promote long-term public health rather than addressing individual symptoms of obesity.

According to WHO, the highest rates of overweight and obesity are observed in countries within the Mediterranean and Eastern Europe.^1^ However, so far, a major proportion of studies on obesogenic neighbourhood factors comes from North American, Australian or Western European countries and cannot be directly applied to the countries of the former Eastern European bloc due to socio-political changes in recent decades, influencing the socio-economic segregation,^17^ characteristics of built environment^18^ and trends in population health.^19^^,^^20^ Therefore, this study aimed to assess and map the obesogenicity of urban neighbourhoods in Brno, the second-largest city in Czechia, and investigate the socio-economic disparities in obesogenic exposures.

## Methods

### Study design and setting

The study was conducted in Brno, the second-largest city in Czechia, with a population of 398 510 residents as of 2021.^21^ The city is organized into 29 self-governing districts, which are then subdivided into 48 administrative parts and further delineated into 296 basic settlement units (BSUs) including, on average, 1346 inhabitants per BSU (ranged from 0 to 12 166 inhabitants).

### Measures

The geospatial data of 12 built environment components previously identified as obesogenic^11^^,^^15^ were gathered for each BSU. Their descriptions and data sources are provided in Table 1. In brief, three components represented food environment (restaurants, food stores, supermarkets), and nine components represented the physical activity environment (sports facilities, sidewalks and cycleway density, intersection density, distance to highway, land-use mix, greenspace density, public transport density, and population density). A zonal statistics function was deployed to assign average density per BSU for each variable.

**Table 1:** Components of the obesogenic index developed for Brno.

Component	Description	Measure	Source, year
Restaurants	Facilities reported in Brno retail research (2021) defined as restaurants, bistros, and cafés.	Kernel density, 1 km search radius	Data Brno^a^ 2021
Food stores	Stores reported in Brno retail research (2021) with an area of ≤400 m^2^ offering specified assortment: all food, fruit and vegetables, meat, fish, or bakery.	Kernel density, 1 km search radius	Data Brno^a^ 2021
Supermarkets	Stores reported in Brno retail research (2021) with an area of >400 m^2^ offering specified assortment: all food, fruit and vegetables, meat, fish, bakery.	Kernel density, 1 km search radius	Data Brno^a^ 2021
Sport facilities	Facilities reported in Brno retail research (2021) defined as sports facilities for racquet sports, gym, fitness, sports hall, leisure centres, other sports facilities.	Kernel density, 1 km search radius	Data Brno^a^ 2021
Sidewalk density	Roads accessible for pedestrians characterized as pedestrian, residential, footway, path, forest track	Line density	OSM 2024
Cycleway density	Roads characterized as cycleways.	Line density	OSM 2024
Intersection density	Density of the intersections of the roads accessible for pedestrians.	Point density	OSM 2024
Distance to highway	Highway was defined as a trunk road.	Euclidean distance	OSM 2024
Land-use mix	Total diversity of land uses.	Entropy index	Urban atlas 2018
Green space	Lands dedicated to forests, green urban spaces, natural grassland, moors.	The proportion of BSU area covered by green space.	Urban atlas 2018
Public transport density	All public transport stops of the Integrated transport system of South-Moravia Region located in Brno.	Point density	Data Brno^a^ 2024
Population density	Usually living population for each BSU based on the data from census 2021.	Ratio of usually living residents to area of the BSU.	Census 2021

OSM, Open Street Map.

a The data portal of the city of Brno (data.brno.cz).

The socio-economic characteristics of each BSU were obtained from the Census 2021. Education was assessed as a continuous variable of proportion of inhabitants with university education. Unemployment was assessed as the proportion of unemployed residents among all potentially economically active residents (individuals aged 15 and older, including those employed for wages, unemployed but actively seeking work, working pensioners, employed students and pupils, and individuals on maternity leave).

### Analysis

**Figure 1:**
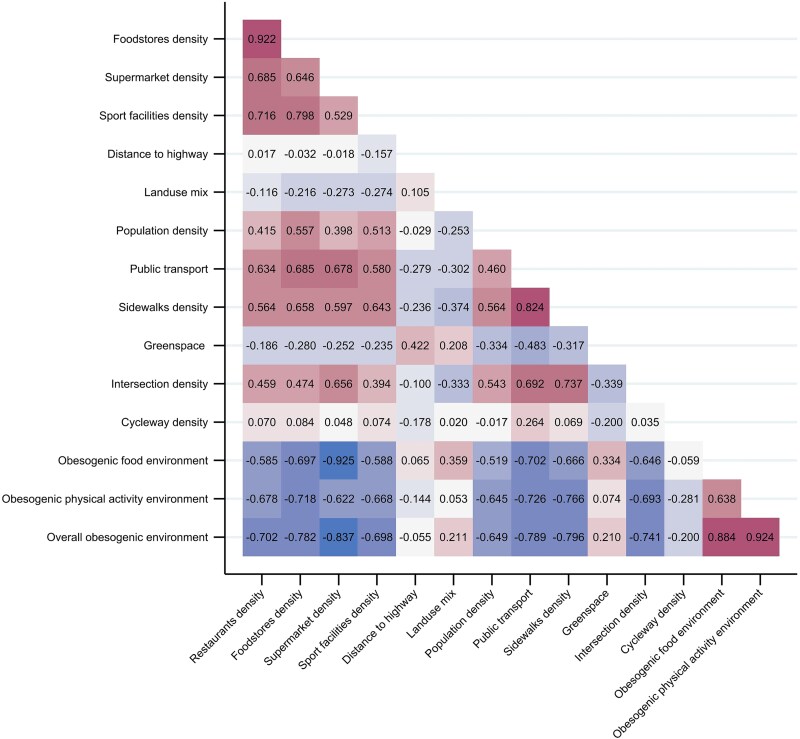
Pearson correlation matrix of obesogenic index components *z*-scores.

The obesogenic index was calculated according to the previously described methodology.^11^ All 12 components were standardized into z-scores. All components except for the density of restaurants were reverse scored so that a higher score corresponded to higher obesogenicity, as theorized in previous studies.^11^ Then, we composed the food and physical activity environmental scores by averaging the z-scores of all relevant components. Further, we calculated the obesogenic index by linear aggregation, taking the arithmetic mean of the food and physical activity environment scores (Supplementary Fig. S1a). The index was then minimum-maximum normalized so that the BSUs under study would range from 0 to 100, with a higher score indicating higher obesogenic potential. Pearson correlations between components *z*-scores and calculated scores were assessed (Fig. 1).

The descriptive statistics at the city level were conducted and the mean, median, range, and interquartile range were reported for socio-economic predictors as well as calculated obesogenic environment scores. The linear regression model was performed to test the associations between socio-economic predictors and obesogenic environment scores. P values less than 0.05 were considered statistically significant.

To assess index sensitivity to weighting methods, we conducted a sensitivity analysis following Lam et al.,^11^ using a hierarchical weighting approach (Supplementary Fig. S1b) and evaluated the consistency of both obesogenic indexes with Pearson correlation. Additionally, global spatial clustering was performed using the global Moran’s Index to detect whether calculated obesogenicity was clustered, dispersed, or randomly distributed across the city. The spatial clustering analysis was performed for the overall obesogenic index as well as separately for food and physical activity environment.

All spatial analyses were done in ArcGIS Pro^22^ and all statistical tests were conducted using STATA^23^ software (version 16.0, StataCorp, College Station, TX, USA).

## Results

### Correlation among environmental components

The correlation matrix (Fig. 1) showed a moderate to strong correlation (*r* = 0.646 to 0.922) among food environment components, with the strongest correlation between restaurants and food stores. For physical components, walkability characteristics (sidewalk density, public transport density, intersection density) showed moderate to strong correlation (*r* = 0.692 to 0.824) which each other and moderate correlations (*r* = 0.394 to 0.646) with sports facilities. Additionally, walkability components and sports facilities were moderate to strongly correlated with all food environment components (Fig. 1).

### Obesogenic environment in Brno

**Figure 2:**
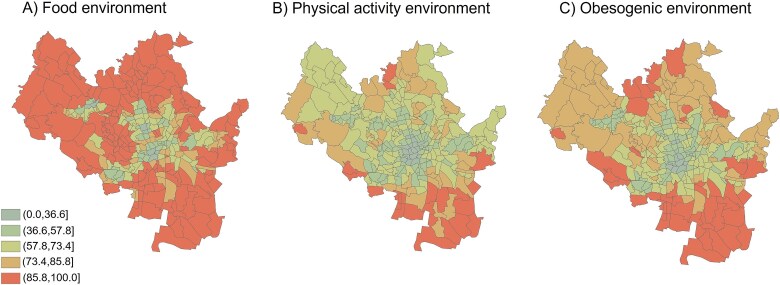
Heatmap of obesogenic food environment (A), physical activity environment (B), and overall obesogenic (C) exposures at the basic settlement’s units of Brno.

The obesogenic environment index was calculated for all 296 BSUs in Brno. By design, the index ranged from 0.00 to 100.00. For food and physical activity environments, the median index scores were 86.55 (IQR = 28.47) and 63.80 (IQR = 20.28), respectively (Table 2). For the overall obesogenic environment, the median index score was 72.09 (IQR = 24.03) (Table 2). Less obesogenic environments tended to concentrate centrally within the city, while higher obesogenicity was found in the peripheries and industrial zones (Fig. 2), as also shown in the spatial cluster analysis (see Supplementary Fig. S2).

**Table 2:** Descriptives of socio-economic predictors and calculated obesogenic environment scores at the BSU level.

	Mean	Median	Range	IQR
**Socio-economic predictors**				
Percentage of inhabitants with university education	32.60	31.92	0.00–66.08	14.55
Unemployment rate	5.46	4.21	0.00–51.56	2.26
**Obesogenic environment scores**				
Food environment^a^	80.16	86.55	0.00–100.00	28.47
Physical activity environment^a^	63.01	63.80	0.00–100.00	20.28
Overall obesogenic environment^a^	69.19	72.09	0.00–100.00	24.03

a Presented scores could range from 0 to 100, with a higher score indicating higher obesogenic potential.

### Social disparities in obesogenic environment

**Figure 3:**
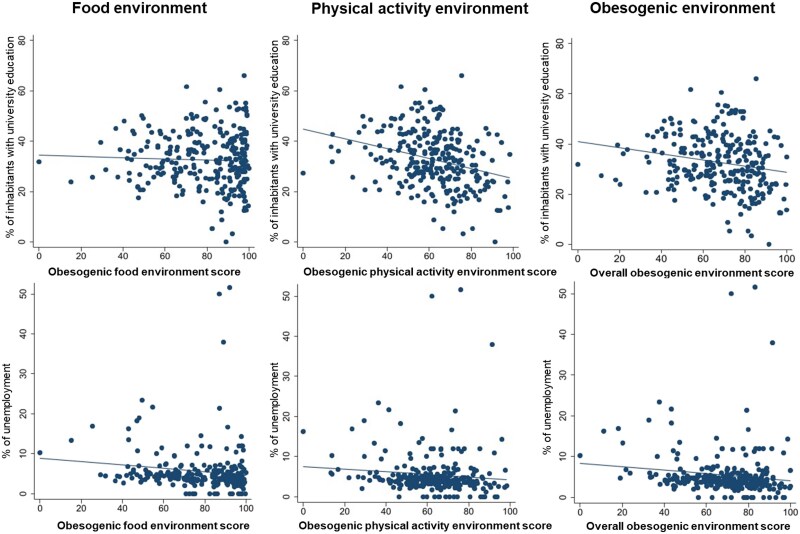
The relationships between socio-economic variables and food, physical activity, and overall obesogenic environment.

We tested the associations between socio-economic variables (unemployment, proportion of people with university education) and obesogenic environment (food, physical activity, and overall) using linear regression analysis. The relationships between variables are visually displayed in Fig. 3. The results showed that a 1% increase in the proportion of university-educated inhabitants was associated with a 0.445 unit decrease in physical activity obesogenic environment scores and a 0.317 unit decrease in overall obesogenic environment scores. Moreover, a 1% increase in the unemployment rate was associated with a 0.474 unit decrease in food obesogenic environment scores and a 0.403 unit decrease in overall obesogenic environment scores (Table 3).

## Discussion

### Main findings of this study

We described the obesogenicity of neighbourhoods in Brno and investigated socio-economic inequalities in these exposures. We calculated three indexes reflecting obesogenic food environment, obesogenic physical activity environment, and overall obesogenic environment. Less obesogenic areas were more centrally located across all assessed environments, likely due to the high walkability and dense concentration of sports facilities in Brno’s city centre. Although the central part of the city is also characterized by a high concentration of restaurants and fast food, considered obesogenic,^15^ they are co-located with other, less unhealthy types of food retail. Regarding socio-economic disparities, at the BSU level, areas with a higher proportion of people with university education tend to show lower obesogenic potential in physical activity environment and overall obesogenic environment. However, we simultaneously observed that localities with higher unemployment exhibited lower obesogenic potential in food and overall obesogenic environment, indicating an unclear socio-economic gradient in Brno.

**Table 3:** The independent associations between socio-economic variables and food, physical activity and overall obesogenic environment.

	Obesogenic food environment^a^		Obesogenic physical activity environment^a^		Overall obesogenic environment^a^	
	b	*P*	b	*P*	b	*P*
Percentage of university education	−0.066	.520	**−0.445**	**<.001**	**−0.317**	**.001**
Percentage of unemployment	**−0.474**	**.017**	−0.281	.110	**−0.403**	**.031**

a The scores range from 0 to 100, with a higher score indicating higher obesogenic potential. Bold values represent statistically significant results *P* < .05.

### What is already known on this topic

The results partially contrast with findings from a Dutch study, which identified the most obesogenic neighbourhoods in city centres and low-SES areas.^11^ Similarly, studies from the USA,^24^ Norway,^16^ the Netherlands,^25^ the UK,^26^ and Canada^27^ found greater fast-food access and fewer grocery stores in disadvantaged neighbourhoods. Additionally, research from Norway^16^ and the USA^28^^,^^29^ reported worse access to sports facilities in neighbourhoods with lower socio-economic status (SES).

When considering the proportion of university-educated inhabitants as an indicator of high neighbourhood SES, our findings, which show higher obesogenic potential in areas with a lower proportion of university-educated residents, are consistent with previous studies. However, when we consider the unemployment rate as an indicator of low SES, our results—indicating higher obesogenic potential in areas with lower unemployment—differ from previous studies, which brings ambiguity into the socio-economic gradient of the obesogenic environment in Brno. These discrepancies may stem from the distinct urban structures in post-communist cities.^30^

During the communist era, cities in Central and Eastern Europe featured compact layouts, large-scale public projects, excess industrial space, and a lack of commercial areas, leading to visual monotony.^31^ In contrast to Western countries, socialist cities exhibited milder symptoms of metropolitan growth. Suburbanization, driven by increasing individualism and growing car dependency, was limited, leading to a more clearly defined urban edge marked by densely constructed prefabricated housing estates.^32^ After the fall of communism, the post-communist cities experienced significant transformations, marked by the transition to market economies, extensive privatization, and suburbanization.^30–32^ This transition may have also contributed to the creation of an obesogenic environment. For example, the shift to market economies resulted in a broader variety of foods in retail, including a wider range of processed items, and facilitated the growth of fast-food outlets.^33^ While the overall availability of various goods has increased, disparities in food retail accessibility have emerged. For instance, large stores may be out of reach for low-income families without cars, forcing them to shop at smaller local stores that often offer fewer food options at higher prices.^33^^,^^34^

In Brno, the lower obesogenicity in the neighbourhoods with higher unemployment rates potentially reflect the specific urban design. The ongoing commercialization of the city centre^30^ has resulted in a high concentration of sports facilities and food retail, which, combined with high walkability, reduce the area’s obesogenic potential. While commercialization may raise unhealthy food outlets, our results suggest this is offset by non-obesogenic features. Along with the rise in commercial use, there has been a corresponding loss of human capital.^35^ Brno’s city centre is experiencing a decline in socio-economic and housing conditions, driven by the social segregation and socialist regime’s neglect of basic maintenance of historical building.^32^ The inconvenient disposition of the historic buildings, mostly constructed in the 19th century, together with its high purchase prices and operating costs fails to appeal to the younger, more affluent generation. Therefore, the central area is facing a population aging as well as decrease in permanent residents, which might lead to rising unemployment.^32^^,^^35^ At the same time, many central areas have undergone revitalization, offering quality housing for individuals with higher SES.^35^ The mixed observations in the socio-economic gradient in our results may, therefore, reflect the diversity of residents in central areas of Brno.

### What this study adds

Our results indicate that the spatial distribution of obesogenic environments may vary by country- or region, potentially influenced by local circumstances. This difference could be related to the previously observed difference in urban social segregation,^36^ however, such evidence is still limited. Likewise, it remains unclear whether the obesogenic potential of environmental elements is consistent especially as most research focuses on North American, Australian or Western European countries, leaving gaps in understanding for Central and Eastern Europe. As such, there is limited evidence about the association between defined components of built environment and obesity in the Czech population. One study conducted by Spilkova in 2016^37^ reported no significant role of the built environment on physical activity and obesity development in the sample of adolescents aged 14 to 15 years. Another study pointed to a relationship between walkability and body fat in young women.^38^

Moving forward, better understanding the combined exposures potentially related to obesity is essential. In Central and Eastern Europe, cultural differences and past socio-political shifts have driven rising obesity rates, highlighting the need for focused research on obesogenic environment. Our study contributes to limited understanding of obesogenic environment in post-communist countries; however, the generalizability of our findings might be constrained. While socialist cities shared common institutional and political influences from centrally driven socialist planning, their socio-spatial patterns were shaped by unique urban conditions, cultural traditions, and socio-economic structures inherited from the past.^30^ Furthermore, the urban transition in post-communist era has been largely shaped by country-specific institutional reforms and shifts in social practices. As a result, significant discrepancies in living standards persist across Central and Eastern Europe,^39^ which might impact social disparities in obesogenic environment. Further studies should focus on the post-communist urban structures in the cities of Central and Eastern Europe and investigate the impact of urban revitalization, gentrification, and socio-economic shifts on obesogenic environments. At the policy level, our findings can inform urban planning strategies that support healthier communities, including zoning regulations, incentives for infrastructure development, and initiatives to improve access to fresh and healthy food. In Brno, our findings highlight the need to invest in peripheral areas such as Bosonohy, Jehnice, Ivanovice, Tuřany, and Žebětín, where walkable access to diverse healthy food options and sports facilities lags behind that of central districts. Generally, in post-communist cities like Brno, it would be beneficial to invest into revitalizing urban spaces to create walkable neighbourhoods that integrate residential, commercial, and recreational spaces. Moreover, investment in public transport networks could improve the connection of and access to underserved areas. Expanding green spaces, pedestrian zones, and community hubs with sports facilities would likely improve walkability and promote active lifestyles, especially in high-density areas with limited recreational space. For public health professionals, it could help to develop precisely targeted health promotion programmes that consider the specific environmental factors influencing a community’s health. Interventions should aim to strengthen individuals’ capacity to cope with obesogenic elements of the built environment while promoting the use of its positive aspects. Given the unclear social gradient observed in current study, further research should employ diverse indicators of socio-economic position (e.g. income, housing condition or financial deprivation) and include individual cohort or registry data which to better understand the relationship between built environment, socio-economic status, and obesity risk in post-communist countries. To enhance the accuracy of assessing obesogenic urban environments, future studies could incorporate a broader range of factors such as safety and aesthetic appeal, significantly influencing urban walkability, or cultural and commercial aspects of the food environment, including portion sizes and food advertising. Moreover, environmental pollutants could be considered, as suggested in previous literature.^40^

### Limitations of this study

The major strength of the current study lies in high-resolution objective measures of the environment. Moreover, to the best of our knowledge, it is first of its kind in Central and Eastern Europe that attempts to quantify obesogenicity of the environment in post-communist areas. We also harnessed detailed administrative data on socio-economic variables. Our study showed contrast on neighbourhood level, but also in terms of clusters of neighbourhoods that could potentially deserve attention for further studies.

However, this study has some limitations which deserve to be mentioned. Firstly, due to availability, geographical data came from different years: from 2018 to 2024. This may have affected the resulting obesogenic index. Previous studies from the Netherlands showed that while food environment or sports facilities might exhibit significant temporal fluctuations,^25^ other components such as walkability and green space remain relatively consistent over time.^41^ Nevertheless, there was no temporal gap between the more dynamic food environmental data and SES data, as they stemmed from the same year (2021). Furthermore, the cross-sectional design and lack of temporal data limit our ability to infer causality or account for potential residential self-selection bias, as individuals may choose to reside in areas that align with their health behaviours. Secondly, the obesogenic index calculation was based on previous study^11^ considering obesogenic components that have been defined mostly in Western studies and may not be transferable to countries with post-communist contexts including Czechia. Moreover, the definitions of obesogenic environment components are primarily derived from studies involving adults, which may reduce their relevance when assessing obesogenic exposure for children. Thirdly, not all previously described components^11^ have been assessed, due to data unavailability which may affect its accuracy, validity, and comparability with other studies. Additionally, the data source used did not account for street food, distinguish between fast food establishments and other types of restaurants, or consider cultural factors in dietary habits, including portion sizes. This may have introduced bias into the assessment of the food environment. Future studies could include street-level audits to capture often-overlooked informal vendors, in collaborations with local authorities and vendor associations to enhance data accuracy. Furthermore, incorporating alternative sources such as crowdsourced platforms, food delivery app data, and business registries could provide a more dynamic and comprehensive view of the food environment.

## Conclusion

In Brno, obesogenic environments varied across neighbourhoods, with lower obesogenicity in central areas due to better walkability and access to sports facilities. Surprisingly, no clear socio-economic gradient was found, as neighbourhoods with higher education levels had lower obesogenicity, similar to areas with higher unemployment. Further research is needed to clarify this gradient in post-communist cities.

## Supplementary Material

Supplementary_materials_fdaf065

## Data Availability

The geographical data underlying this article are available from Data Hub Brno (https://datahub.brno.cz/), OpenStreetMap (https://www.openstreetmap.org/) and Urban Atlas (10.2909/fb4dffa1-6ceb-4cc0-8372-1ed354c285e6). Socioeconomic data from Census were provided by Brno City Municipality with permission and are available upon request to the Municipality or the Czech Statistical Office.

## Notes

**Anna Bartoskova Polcrova**, Researcher

**Thao Minh Lam**, Researcher

**Hynek Pikhart**, Professor

**Jeroen Lakerveld**, Associate professor
