# Supplementary material for: Social disparities in exposures to neighbourhood obesogenic built environments in Czechia
Source: J Public Health (Oxf). 2025 Jun 9;47(3):436–45. doi: 10.1093/pubmed/fdaf065 (PMC12395951; doi:10.1093/pubmed/fdaf065)
Supplement: Supplementary_materials_fdaf065 [file supplementary_materials_fdaf065.docx]

**Supplementary materials**

**Social disparities in exposures to neighbourhood obesogenic built environments in Czechia**

Anna Bartoskova Polcrova^1^, Thao Minh Lam^2,3,4^, Hynek Pikhart^1,5^, Jeroen Lakerveld ^2,3,4^

^1^RECETOX, Faculty of Science, Masaryk University, Kotlarska 2, Brno, Czech Republic

^2^Department of Epidemiology and Data Science, Amsterdam University Medical Centers, Vrije Universiteit Amsterdam, Amsterdam, the Netherlands

^3^Amsterdam Public Health, Health Behaviours and Chronic Diseases, Amsterdam, the

Netherlands

^4^Upstream Team, Amsterdam University Medical Centers, Vrije Universiteit Amsterdam, Amsterdam, the Netherlands

^5^Department of Epidemiology and Public Health, University College London, United Kingdom

Supplementary figure 1: Equations for obesogenic index calculations

**
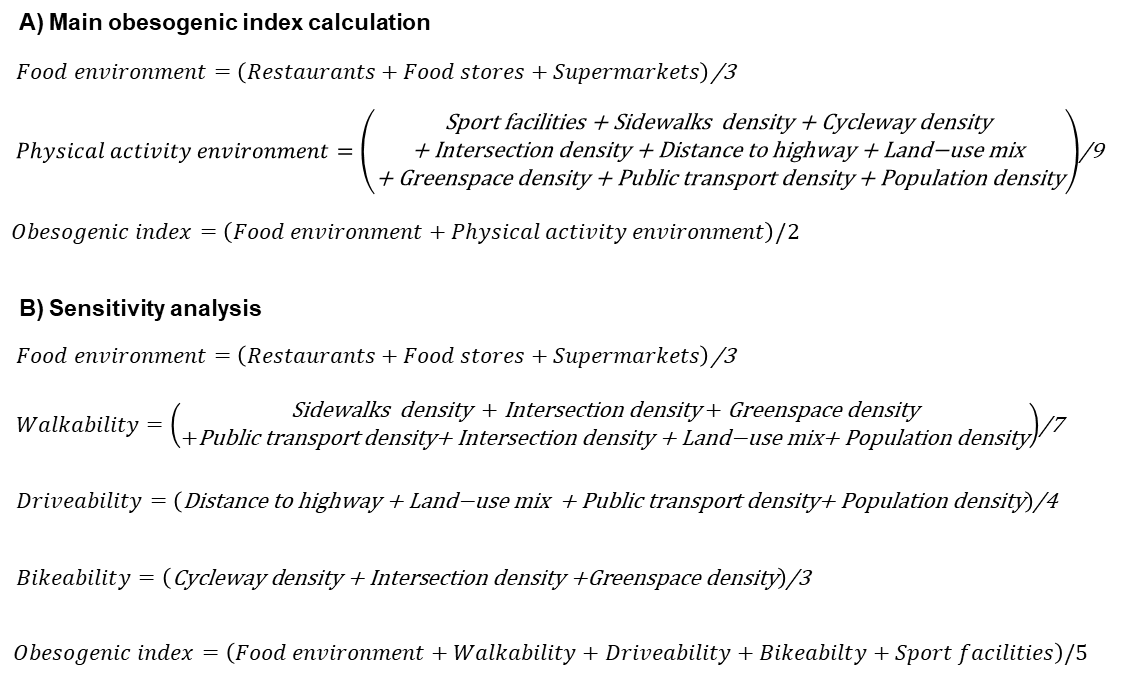
**


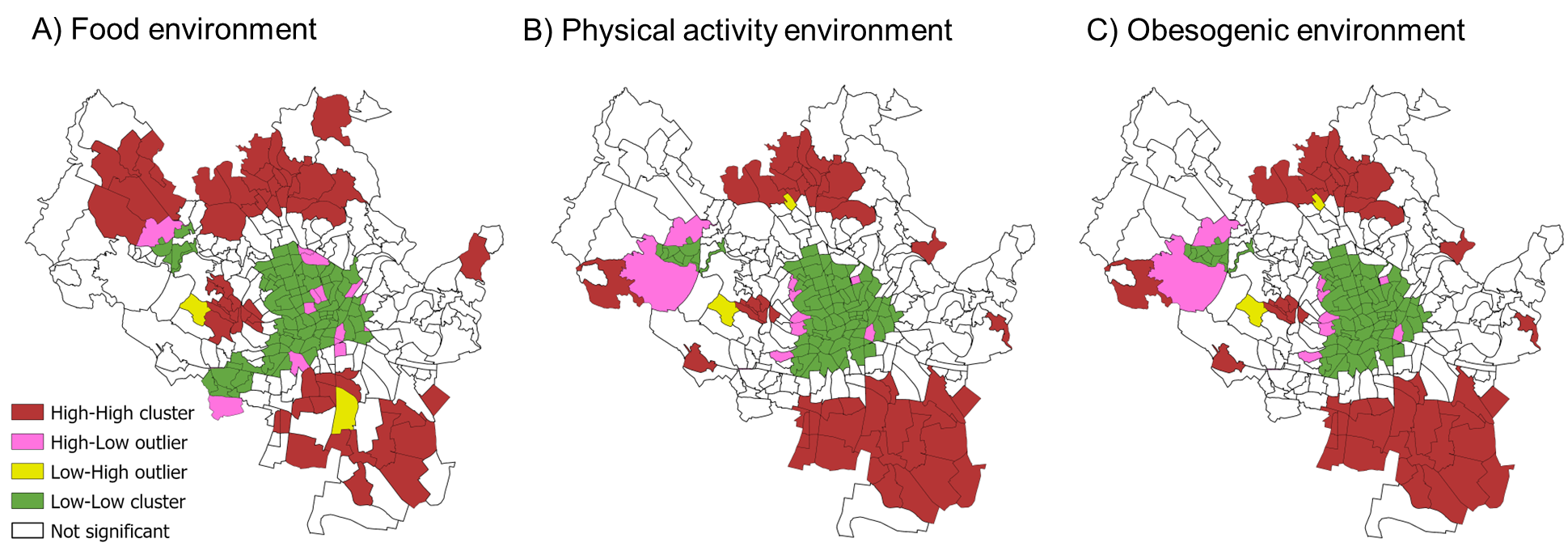
Supplementary figure 2: Spatial cluster analysis of obesogenic food, physical activity and overall obesogenic exposures. High-high and low-low clusters represent areas with high or low scores, surrounded by areas with similarly high or low scores. High-low outliers are areas with high scores surrounded by low-scoring areas, while low-high outliers have low scores surrounded by high-scoring areas.
